# The First-Known Case of Hereditary Heterozygous Butyrylcholinesterase Deficiency in a Patient on Dialysis

**DOI:** 10.7759/cureus.53153

**Published:** 2024-01-29

**Authors:** Naoki Tokunaga, Hisato Shima, Takuya Okamoto, Masato Maekawa, Jun Minakuchi

**Affiliations:** 1 Laboratory, Kawashima Hospital, Tokushima, JPN; 2 Kidney Disease, Kawashima Hospital, Tokushima, JPN; 3 Laboratory Medicine, Hamamatsu University School of Medicine, Hamamatsu, JPN

**Keywords:** protein-energy wasting, liver dysfunction, albumin, dialysis, hereditary deficiency, butyrylcholinesterase

## Abstract

Serum levels of butyrylcholinesterase (BChE) are commonly used to assess liver function. Its levels have been reported to be significantly lower in patients undergoing dialysis. To the best of our knowledge, this is the first report of hereditary heterozygous BChE deficiency in a patient undergoing dialysis. Medical staff involved in the care of patients with BChE deficiency should be aware of anesthetic usage, because prolonged neuromuscular paralysis following the administration of succinylcholine or mivacurium may occur. However, in the heterozygotes, BChE activity is not completely absent. Therefore, differentiating patients undergoing dialysis is challenging. A 52-year-old man underwent living-related kidney transplantation for focal segmental glomerulosclerosis at 22 years of age. As the renal function gradually worsened, the patient began to receive combined hemodialysis and peritoneal dialysis therapy. No problems with anesthesia were observed in past surgeries. The patient’s BChE levels fluctuated between 76 and 170 U/L (reference range: 198-495 U/L); however, they had never been previously investigated. We suspected hereditary heterozygous BChE deficiency because the patient’s sister was also diagnosed with it. DNA sequencing revealed a heterozygous missense mutation (Gly365Arg) and a K-variant (Ala539Thr). Patients on dialysis with low serum BChE levels often present with low albumin levels which may be overlooked as malnutrition. Thus, BChE deficiency should be suspected in patients on dialysis with unexplained low serum BChE levels. In the case of heterozygous BChE deficiency, the reference value is low, and continuous monitoring is crucial.

## Introduction

Human tissues have two types of cholinesterase activities: acetylcholinesterase (AChE) and butyrylcholinesterase (BChE) [1]. AChE functions in the transmission of nerve impulses and is one of the most efficient enzymes in the central and peripheral nervous systems [1]. On the other hand, the physiological role of BChE remains unknown [1]. BChE is synthesized in the liver, and because its half-life (approximately 10 days) is shorter than that of albumin, serum BChE level is a sensitive marker for protein synthesis. Additionally, it is a useful marker of nutritional status and prognosis [2]. The reference interval of BChE is from 198 to 495 U/L and decreases in various pathological conditions such as chronic liver disease, liver cirrhosis, severe liver injury, malnutrition, chronic infection, myocardial infarction, renal failure, and malignancy [3,4]. In addition to the external factors, hereditary BChE deficiency should be suspected as a cause of low BChE activity. Patients with a BChE deficiency are not at risk in daily life. However, a risk of protracted apnea syndrome exists due to the delayed decomposition of succinylcholine or mivacurium, muscle relaxants used during surgery [5]. If an ester-type local anesthetic is administered, the blood concentration of the administered drug may induce convulsions [6]. However, diagnosing hereditary heterozygous BChE deficiency in patients on dialysis is challenging because their serum BChE level is lower than that in healthy individuals due to various factors such as an increase in homocysteine levels and oxidative stress [7]. Herein, we report the first case of a man on dialysis with a hereditary heterozygous BChE deficiency. This report provides new clues for the diagnosis of hereditary heterozygous BChE deficiency in patients on dialysis.

## Case presentation

A 52-year-old man was treated with combined hemodialysis (HD) and peritoneal dialysis (PD) at our hospital. The patient underwent living-related kidney transplantation from his mother because of focal segmental glomerulosclerosis at 22 years of age. As the renal function gradually worsened, the patient was started on PD and combined therapy at the ages of 48 and 49 years, respectively. No problems existed with anesthesia in past surgeries. Laboratory data for aspartate and alanine aminotransferase, serum total cholesterol, and triglyceride were within the reference interval. Serum BChE levels had fluctuated between 76 and 170 U/L, while serum albumin levels were between 2.6 and 3.6 g/dL for about four years after starting PD and HD combined therapy. However, the patient had never been previously investigated or followed up.

**Figure 1:**
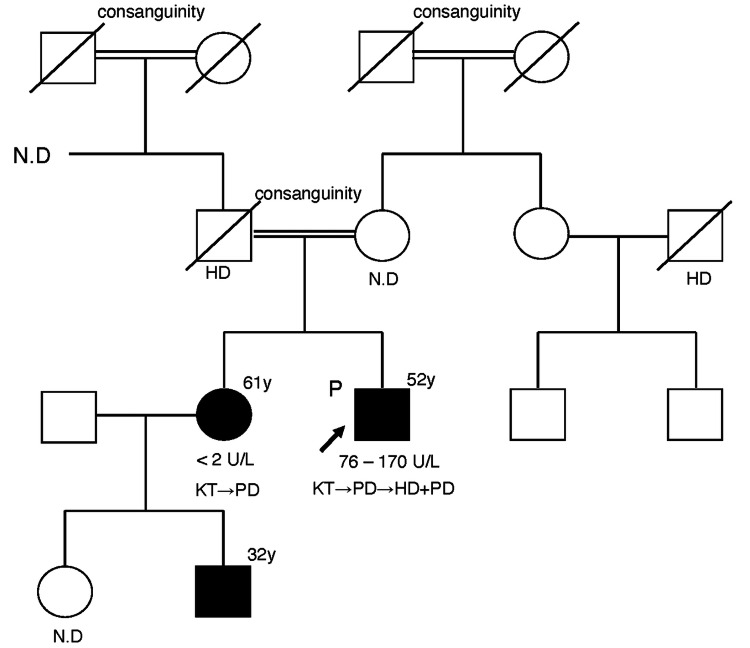
Family pedigree Serum BChE levels were 76-170 U/L and extremely low (<2 U/L), respectively P■: proband; ●: sister (affected person); ■: nephew (affected person); HD: hemodialysis; PD: peritoneal dialysis; KT: kidney transplantation; N.D: no data; BChE: butyrylcholinesterase

We recently reported the case of a 61-year-old woman who was a renal transplant recipient and who was the older sister of the man. The woman complained of diarrhea, anorexia, and headache during oral steroid treatment, causing adrenal insufficiency associated with steroid malabsorption [8]. The family pedigree is shown in Fig. 1.

**Figure 2:**
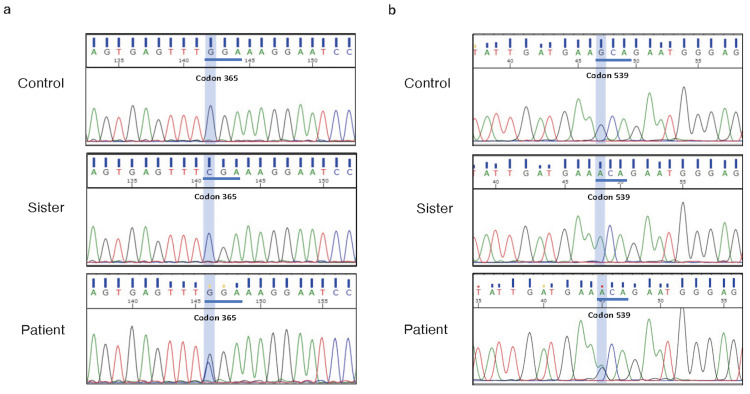
DNA sequencing analysis (a) DNA sequencing analysis confirmed homozygous (sister) and heterozygous (patient) missense mutations (1093G > A, leading to Gly365Arg). (b) Homozygous (sister) and heterozygous (patient) K-variants are also revealed (1615G > A, leading to Ala539Thr)

The woman’s serum BChE level was extremely low (<2 U/L). Thus, we suspected the sister of having hereditary BChE deficiency and performed DNA sequencing analysis, which revealed a homozygous missense mutation (1093G > A, leading to Gly365Arg) and a K-variant (1615G > A, leading to Ala539Thr) (Fig. 2a, 2b). Therefore, we suspected the man to also have a hereditary BChE deficiency. DNA sequencing revealed that the man was heterozygous for the same mutation and a K-variant (Fig. 2a, 2b).

Additionally, we detected the same mutation in the man’s nephew (data not shown). There are no treatment options available. Therefore, we have routinely monitored liver function along with serum BChE levels.

## Discussion

To the best of our knowledge, this is the first report of hereditary BChE deficiency in a patient undergoing dialysis. This study highlights an important clinical issue that patients on dialysis with hereditary heterozygous BChE deficiency may frequently be missed.

Hereditary BChE deficiency is classified as a pharmacogenetic disease with an autosomal recessive inheritance [5]. The *BCHE* gene is located on chromosome 3q26 and comprises four exons, occupying approximately 80% of exon 2 [9]. Regarding genetic polymorphisms, dibucaine-resistant (type A) and fluoride-resistant (type F) variants are common in Europe and the United States, both of which are rare in Japan [10]. In Japan, several silent types (type S) exist that lead to a decrease in BChE activity. In Japan, the frequency of hereditary BChE deficiency is one in 150-200 individuals for heterozygotes and one in 100,000-150,000 individuals for homozygotes. In this study, the man on dialysis had a G365R mutation and a K-variant, both of which are common in Japan. The G365R mutation, the most common type S mutation in Japan, is present in approximately 0.2% of cases and leads to decreased BChE [10]. Contrastingly, the K-variant is present in approximately 18% of the cases in Japan and reduces BChE activity by approximately 30% [11]. Furthermore, this mutation is associated with the G365R mutation [11]. 

Though there may be no clinical problem with BChE deficiency, two main reasons exist for a definitive diagnosis of the genetic defects involving BChE. Firstly, patients diagnosed with BChE deficiency should inform their doctors about their condition before surgery. Medical staff involved in the care of patients with BChE deficiency should be aware of anesthetic usage. We consider that the serum BChE level in this case was high enough not to induce anesthetic problems. Secondly, patients with BChE deficiency are often suspected of having hepatic dysfunction. There are no treatment options available. However, once diagnosed with hereditary BChE deficiency, low serum BChE levels should be considered as a baseline, and liver function should be routinely monitored.

By means of searching PubMed, we found 44 cases of BChE deficiency since 2010. Of the 44 cases, 23 were due to genetic causes. The most common symptoms were prolonged paralysis and apnea (37 cases). Four were asymptomatic. All cases were nondialyzed. To the best of our knowledge, our case is the first report of hereditary heterozygous BChE deficiency in a patient undergoing dialysis. The patient was asymptomatic and diagnosed with hereditary heterozygous BChE deficiency with the diagnosis of the patient’s sister. Patients on dialysis experience a high proportion of protein-energy wasting due to uremic substance accumulation and increased protein catabolism, regardless of nutritional intake [12]. Moreover, considering various factors including albumin leakage due to dialysis, BChE levels are preferable for evaluating protein synthesis [13,14]. Previous reports suggested that the BChE level was reduced and an independent prognostic factor in patients on dialysis [15-17]. Considering the frequency of hereditary heterozygous BChE deficiency, the number of patients on dialysis diagnosed is thought to be very small. BChE activity was not completely absent in heterozygotes. Therefore, differentiating patients on dialysis with BChE deficiency is challenging, and it may be overlooked as malnutrition. There are several limitations in this study. Firstly, the patient and his sister have developed end-stage kidney disease. However, the relationship between hereditary BChE deficiency and primary causes of end-stage kidney disease is unclear. Secondly, it is necessary to develop a useful method for distinguishing the hereditary causes of low serum BChE levels.

## Conclusions

In the case of heterozygous BChE deficiency, the reference value might be lower than the normal lower limit. Patients on dialysis with low serum BChE levels often present with low albumin levels which may be overlooked as malnutrition. Therefore, BChE deficiency should be suspected in patients on dialysis with unexplained low serum BChE levels, and continuous monitoring is crucial.
